# Effect of anesthetics on postoperative nausea and vomiting after peripheral vascular surgery in end-stage renal disease patients: A retrospective observational study

**DOI:** 10.3389/fsurg.2022.1054670

**Published:** 2022-11-23

**Authors:** Ho Bum Cho, Sun Young Park, Nayoung Kim, Sang Jin Choi, Sanghoon Song, Jae Hwa Yoo, Mun Gyu Kim, Ji Won Chung

**Affiliations:** Department of Anesthesiology and Pain Medicine, Soonchunhyang University Hospital Seoul, Seoul, South Korea

**Keywords:** ESRD, PONV, peripheral vascular surgery, propofol, TIVA

## Abstract

**Background:**

Propofol-based total intravenous anesthesia (TIVA) is considered a prophylactic approach to decrease postoperative nausea and vomiting (PONV). Despite general anesthesia commonly being performed in end-stage renal disease (ESRD) patients, PONV in ESRD patients has not been well-described. We investigated PONV in peripheral vascular surgery under general anesthesia in ESRD patients.

**Methods:**

To compare PONV between propofol-based TIVA and anesthesia with volatile anesthetics, we collected retrospective data from patients who underwent peripheral vascular surgery under general anesthesia from July 2018 to April 2020. We performed univariable and multivariable analyses, including factors that could be associated with PONV and those previously shown to affect PONV.

**Result:**

A total of 1,699 peripheral vascular surgeries under general anesthesia in ESRD patients were eligible for analysis. Based on the multivariable analysis, TIVA (odds ratio [OR], 0.45; 95% confidence interval [CI], 0.35–0.60; *P* < 0.001) significantly decreased PONV. Female sex (OR, 1.85; 95% CI, 1.44–2.38; *P* < 0.001) and anesthetic duration (OR, 1.01; 95% CI, 1.00–1.01; *P* < 0.001) were associated with increased PONV.

**Conclusion:**

Propofol-based TIVA is the most influential factor decreasing PONV after peripheral vascular surgery in ESRD patients. Anesthesiologists can apply propofol-based TIVA as an alternative to anesthesia with volatile anesthetics.

## Introduction

Postoperative nausea and vomiting (PONV) is one of the most common adverse effects of general anesthesia (1). Generally, the importance of PONV has been devalued, although it has a significant impact on postoperative care. PONV can delay discharge, disrupt oral intake, and lead to serious complications such as wound dehiscence and anatomic leaks. Therefore, it can increase treatment costs (2). Furthermore, PONV is a more common cause of patient discomfort than postoperative pain (3). Numerous factors affect the incidence of PONV, including patient characteristics, anesthetic factors, and surgical procedures (4). Among the prophylactic options for PONV, propofol-based total intravenous anesthesia (TIVA) is considered an excellent anesthetic strategy (5).

The incidence of end-stage renal disease (ESRD), the final stage of chronic kidney disease (CKD), is increasing globally. In the year 2000, approximately 1.1 million patients worldwide were being treated for CKD, showing an increase of 6%–7%, which is greater than the global population growth rate. The number of hemodialysis patients is estimated to reach 3,500,000 by 2020 (6). For these patients, hemodialysis is the most common treatment, which has increased the survival rate and improved patient quality of life (7). To achieve vascular access for chronic hemodialysis, peripheral vascular surgeries are performed in ESRD patients (8).

Although vascular access surgery (arteriovenous fistula formation) for hemodialysis can be performed under local anesthesia alone, many patients require general anesthesia for complicated peripheral vascular surgeries (e.g., graft interposition or aneurysm removal) due to the complexity of the procedures. For this reason, general anesthesia is commonly performed in ESRD patients.

Maintenance of general anesthesia should be achieved using short-acting drugs with minimal renal metabolism. Generally, short-acting volatile anesthetics such as desflurane or sevoflurane are preferred and the opiate remifentanil and the hypnotic propofol can be administered through continuous intravenous infusion as an alternative (9, 10). However, volatile agents are commonly considered the main cause of PONV, whereas TIVA with propofol is thought to decrease PONV (5, 11). Furthermore, there is a relatively high incidence of nausea and vomiting in hemodialysis patients (6). Nevertheless, PONV in peripheral vascular surgery for ESRD patients has not been well-described.

The aims of this study were to investigate PONV in peripheral vascular surgery under general anesthesia in ESRD patients and to compare the incidence between propofol-based TIVA and anesthesia with volatile anesthetics.

## Materials and methods

To compare PONV after general anesthesia in ESRD patients with propofol-based TIVA or anesthesia with volatile anesthetics, retrospective data collection was performed from July 2018 to April 2020 at Soonchunhyang University Hospital, Seoul, Republic of Korea. This retrospective observational study was approved by Soonchunhyang University Hospital's institutional review board (IRB number: SCHUH2020-06-004). Written informed consent was waived because of the retrospective case-control nature of the study. Our findings are presented following the format recommended by the Strengthening the Reporting of Observational Studies in Epidemiology guidelines (12). All methods were carried out in accordance with relevant guidelines and regulations.

## Study population

We retrospectively enrolled 1,923 consecutive cases: all were ESRD patients who underwent peripheral vascular surgery under general anesthesia at age 30–90 years. Among them, emergency surgeries and cases without postoperative visit records for managing PONV were excluded.

## Data collection

Medical records were reviewed retrospectively for patient characteristics, laboratory data, medical treatments, and clinical outcomes. We defined PONV as any nausea, retching, or vomiting according to the postoperative visit records. Demand for antiemetics and medical records indicating PONV in the post-anesthesia care unit (PACU) on postoperative day (POD) 1 were analyzed.

## Anesthetic management

When departing for the operating theater, all patients were premedicated with 0.1 mg of glycopyrrolate intramuscularly, except when contraindicated. Upon arrival in the operating theater, standard monitoring devices were applied, including electrocardiography, pulse oximetry, and an oscillometric noninvasive blood-pressure cuff. Bispectral index monitoring (BIS System; Aspect Medical Systems, Newton, MA, United States) was performed for all participants.

In the TIVA group, general anesthesia was induced and maintained with propofol and remifentanil *via* effect site targeting using a target-controlled infusion system (Orchestra Primea; Fresenius Kabi AG, Bad Homburg, Germany) after intravenous lidocaine (40 mg) administration. Propofol was administered using the Schnider pharmacokinetic model and remifentanil using the Minto model. The target concentrations of propofol and remifentanil were maintained at 2–5 μg/ml and 0–6 ng/ml, respectively, according to a BIS of 40–60.

In the volatile-anesthetics group, induction was performed using intravenous lidocaine (40 mg), propofol (1–1.5 mg/kg), and rocuronium (0.6 mg/kg) for neuromuscular blockade. Anesthesia was maintained with oxygen, medical air, and volatile anesthetics, including desflurane (*n* = 170, 29.1%) or sevoflurane (*n* = 415, 70.9%). Patients were administered intravenous remifentanil as required in the same way as the TIVA group. The volatile anesthetic and remifentanil dose were adjusted to achieve target BIS values of 40–60.

Patients received intravenous ephedrine (4 mg), phenylephrine (50 μg), or an inotropic infusion as required for blood pressure values below 20% of baseline during the operation. At skin closure in both groups, patients received intravenous fentanyl (0.3–0.5 µg/kg). At the end of the surgical procedure, the neuromuscular blockade was reversed with intravenous pyridostigmine (0.2 mg/kg) and glycopyrrolate (5 µg/kg) or with sugammadex (1–2 mg/kg) as needed. Tracheal extubation was performed under a monitoring train-of-four ratio >0.9.

The agents used for anesthesia depended on the discretion of the anesthesiologist assigned to each case. In the PACU and ward, patients received opioid or anti-emetics on demand.

## Statistical analysis

The Kolmogorov-Smirnov test was used to test the hypothesis of a normal distribution for continuous variables. All continuous variables were reported as means ± standard deviations and all categorical variables were reported as n values (proportion, %). Categorical variables were compared using the chi-square test or Fisher's exact test, and continuous variables were compared using the *t*-test or Mann-Whitney *U* test for intergroup comparisons of PONV and other clinical variables. To explore the relationships between PONV and other clinical variables, we performed univariable and multivariable analyses, including factors that could be associated with PONV and those previously known to have an effect on PONV. R software (version 4.0.0; April 24, 2020, R Foundation for Statistical Computing, Vienna, Austria) was used for all statistical analyses; a *P*-value <0.05 was considered significant.

## Results

Among 1,923 cases, 224 were excluded because of a lack of postoperative visit records or emergency surgery. A total of 1,699 peripheral vascular surgeries under general anesthesia in ESRD patients were identified during the study period and all were included in the analysis.

## Patient characteristics

The clinical characteristics of the patients are presented in Table 1. Age (*P* < 0.001), hypertension (*P* < 0.001), and diabetes mellitus (*P* < 0.001) were higher in the TIVA group than in the volatile group. The proportion of female sex (*P* < 0.001) and previous cerebrovascular accidents were higher in the volatile group (*P* = 0.02) than in the TIVA group. There were no significant differences in atrial fibrillation, current angina, previous myocardial infarction, asthma, chronic obstructive lung disease, or obesity between the two groups.

**Table 1:** Clinical patient characteristics.

Characteristics	Total (*n* = 1,699)	TIVA group (*n* = 1,114)	Volatile group (*n* = 585)	*P*-value*
Sex (M: F)	860 (50.62%): 839 (49.38%)	613 (55.03%): 501 (44.97%)	247 (42.22%): 338 (57.78%)	<0.001
Age (years)	63.89 ± 13.62	65.1 ± 13.08	61.59 ± 14.35	<0.001^†^
Hypertension	1,405 (82.7%)	952 (85.46%)	453 (77.44%)	<0.001
Atrial fibrillation	90 (5.3%)	54 (4.85%)	36 (6.15%)	0.30
Current angina	99 (5.83%)	61 (5.48%)	38 (6.5%)	0.46
Previous MI	51 (3%)	29 (2.6%)	22 (3.76%)	0.24
Diabetes mellitus	845 (49.74%)	597 (53.59%)	248 (42.39%)	<0.001
Previous CVA	236 (13.89%)	139 (12.48%)	97 (16.58%)	0.02
Asthma	31 (1.82%)	19 (1.71%)	12 (2.05%)	0.75
COPD	15 (0.88%)	11 (0.99%)	4 (0.68%)	0.72
Obesity	6 (0.35%)	4 (0.36%)	2 (0.34%)	>0.99^‡^

COPD, chronic obstructive pulmonary disease; CVA, cerebrovascular accident; MI, myocardial infarction.

All continuous variables are reported as the mean ± SD and all categorical variables as *n* (proportion, %). Data were analyzed using the ^†^*t*-test, *χ*^2^ test, and ^‡^Fisher's exact test.

Obesity defined as BMI > 25 (kg·m^−2^).

* *P*-value for an analysis between the TIVA group and volatile group.

## Anesthetic management and PONV

**Figure 1:**
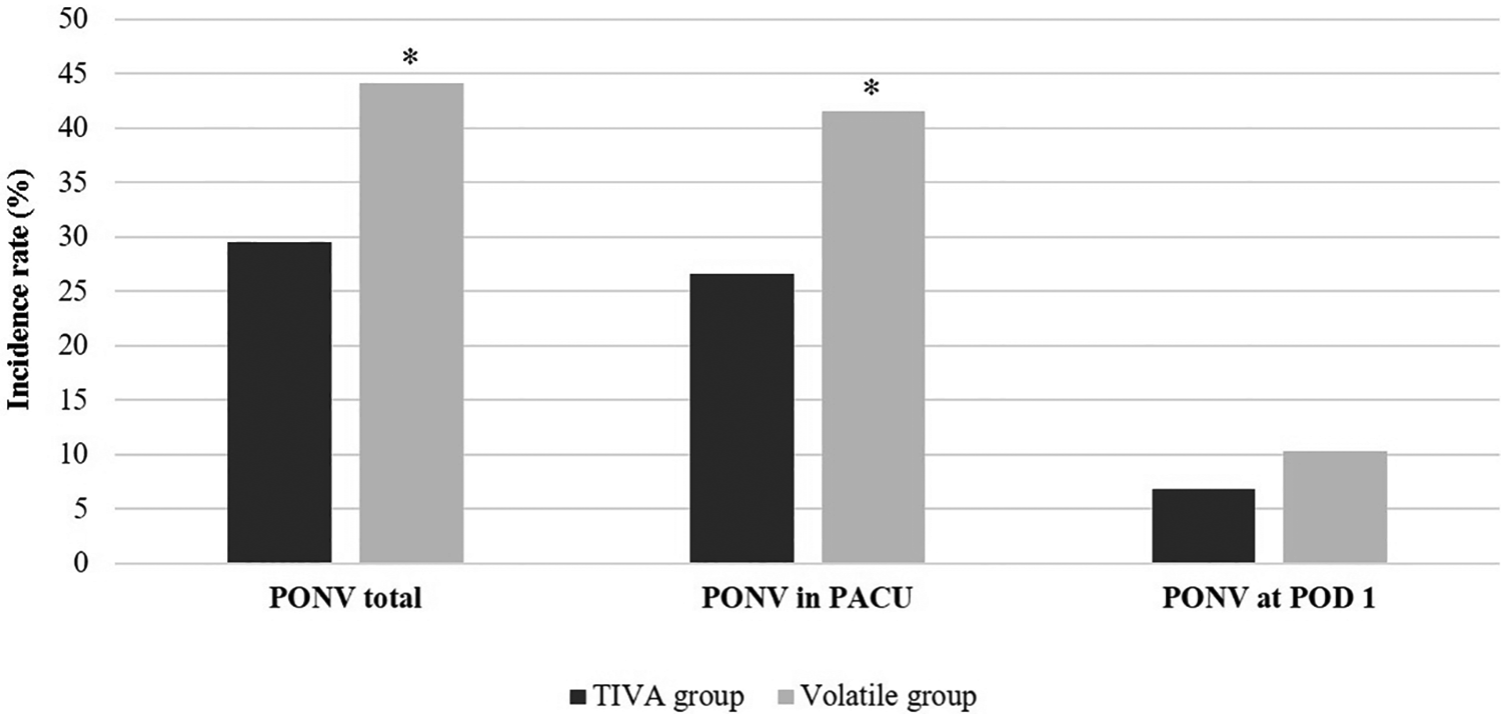
Incidence rate of postoperative nausea and vomiting (PONV) between total intravenous anesthesia (TIVA) group and volatile anesthesia group. *P value <0.05 between two groups.

Table 2 shows anesthetic management and PONV. The incidence of PONV was significantly higher in the volatile group in total (*P* < 0.001) and in the PACU (*P* < 0.001) than in the TIVA group (Figure 1). Anesthetic duration (*P* < 0.001) and the volume of intraoperative crystalloid infusion (*P* < 0.001) were higher in the volatile group than in the TIVA group. PONV at POD 1, use of intraoperative vasoactive agents, use of postoperative inotropic agents, laryngeal mask airway, patient-controlled analgesia (PCA), fentanyl dosage in PCA, and dosage of administered antiemetics (palonosetron hydrochloride and ramosetron hydrochloride) did not differ significantly between the two groups.

**Table 2:** Anesthetic management and PONV.

	Total (*n* = 1,699)	TIVA group (*n* = 1,114)	Volatile group (*n* = 585)	*P*-value*
**Anesthetic duration (min)**	116.83 ± 49.39	106.15 ± 43.08	137.17 ± 54.08	<0.001^†^
**Intraoperative vasoactive agents**				
Ephedrine	644 (37.9%)	417 (37.4%)	228 (38.9%)	0.746
Phenylephrine	411 (24.1%)	253 (22.7%)	158 (27.0%)	0.333
Dopamine infusion	19 (1.1%)	15 (1.4%)	4 (0.7%)	0.495
Norepinephrine infusion	19 (1.1%)	10 (0.9%)	9 (1.5%)	0.556
Crystalloid	172.66 ± 195.1	164.61 ± 194.6	187.99 ± 195.3	<0.001^†^
LMA	435 (25.6%)	285 (25.58%)	150 (25.64%)	>0.99
PCA	273 (14.20%)	189 (16.97%)	84 (14.36%)	0.13
Fentanyl dosage in PCA	1,071.79 ± 291.25	1,053.44 ± 307.10	1,113.10 ± 248.75	0.21^†^
Palonosetron HCl	0.15 ± 0.03	0.14 ± 0.05	0.15 ± 0	0.41^₭^
Ramosetron HCl	0.71 ± 18	0.72 ± 0.17	0.7 ± 0.18	0.82^†^
PONV total	587 (34.55%)	329 (29.53%)	258 (44.1%)	<0.001
PONV in PACU	539 (31.72%)	296 (26.57%)	243 (41.54%)	<0.001
PONV at POD 1	96 (7.80%)	61 (6.86%)	35 (10.26%)	0.06

HCl, hydrochloride; LMA, laryngeal mask airway; PACU, post-anesthesia care unit; PCA, patient-controlled analgesia; POD, postoperative day; PONV, postoperative nausea and vomiting.

All continuous variables are reported as mean ± SD and all categorical variables as *n* (proportion, %). Data were analyzed using the ^†^*t*-test, ^₭^Mann–Whitney *U* test, and *χ*^2^ test.

* *P*-value for an analysis between the TIVA group and volatile group.

## Univariable and multivariable analyses of factors associated with PONV

Based on our univariable analysis, TIVA (*P* < 0.001), female sex (*P* < 0.001), age (*P* = 0.001), anesthetic duration (*P* < 0.001), fentanyl dosage in PCA (*P* < 0.001), and volume of intraoperative crystalloid infusion (*P* = 0.01) were significant factors affecting PONV (Table 3). Among these factors, TIVA (odds ratio [OR], 0.53; 95% confidence interval [CI], 0.43–0.65) and age (OR: 0.99; 95% CI, 0.98–1.00) were associated with decreased PONV. Female sex (OR, 1.85; 95% CI, 1.78–2.69) and anesthetic duration (OR, 1.01; 95% CI, 1.01–1.01) were associated with increased PONV.

**Table 3:** Univariable and multivariable analysis of factors associated with PONV.

	Univariable analysis		Multivariable analysis	
	OR (95% CI)	*P*-value	OR (95% CI)	*P*-value
TIVA	0.53 (0.43–0.65)	<0.001	0.45 (0.35–0.60)	<0.001
Sex (F)	2.19 (1.78–2.69)	<0.001	1.85 (1.44–2.38)	<0.001
Age (years)	0.99 (0.98–1.00)	0.001	1.00 (0.9–1.01)	0.42
Hypertension	1.07 (0.82–1.39)	0.63	1.41 (0.98–2.03)	0.07
Previous MI	1.23 (0.69–2.18)	0.48	1.22 (0.61–2.42)	0.58
Diabetes mellitus	0.84 (0.69–1.02)	0.08	0.95 (0.73–1.25)	0.74
Previous CVA	1.17 (0.88–1.56)	0.27	1.04 (0.72–1.51)	0.82
Anesthetic duration (min)	1.01 (1.01–1.01)	<0.001	1.01 (1.00–1.01)	<0.001
Fentanyl dosage in PCA	1.00 (1.00–1.00)	<0.001	1.00 (1.00–1.00)	<0.001
Crystalloid	1.00 (1.00–1.00)	0.01	1.00 (1.00–1.00)	0.94

CI, confidence interval; CVA, cerebrovascular accident; MI, myocardial infarction; OR, odds ratio; PCA, patient-controlled analgesia; TIVA, total-intravenous anesthesia.

Wald confidence intervals were calculated.

Based on our multivariable analysis, TIVA (*P* < 0.001), female sex (*P* < 0.001), anesthetic duration (*P* < 0.001), and fentanyl dosage in PCA (*P* < 0.001) were significant factors affecting PONV. Multivariable analysis showed that TIVA (OR, 0.45; 95% CI, 0.35–0.60) decreased PONV. Female sex (OR, 1.85; 95% CI, 1.44–2.38) and anesthetic duration (OR, 1.01; 95% CI, 1.00–1.01) were associated with increased PONV.

## Discussion

In this retrospective observational study, TIVA was the most influential factor decreasing PONV after peripheral vascular surgery in ESRD patients. Female sex and anesthetic duration were factors that increasing PONV. The total incidence of PONV was 34.55%. Our study shows that propofol-based TIVA could be considered an alternative anesthetic method to reduce PONV in peripheral vascular surgery for ESRD patients.

Several independent factors are thought to be associated with PONV. These factors can be divided into multiple categories, including patient-specific (age, sex, smoking status, history of motion sickness or previous PONV), anesthetic (volatile anesthetics, intraoperative use of opioids, hydration, anesthetic duration), surgical (type and postoperative use of opioid), and other (mask ventilation, body mass index, pain) (4, 13). Among these, the most reliable risk factors of PONV were female sex, history of PONV or motion sickness, non-smoker, younger age, volatile anesthetics and postoperative opioids (14).

In ESRD patients, previous studies reported a higher prevalence of upper gastrointestinal (GI) symptoms such as nausea (74%), vomiting (68%), and anorexia (64%) (15). The reason for the high prevalence of GI symptoms in ESRD patients is unclear. Nevertheless, multiple etiologies such as treatments for the digestive system, the patient's diet, medication regimen, and developed disabilities are considered major causes of nausea and vomiting (16, 17).

Among the available anesthetics, propofol is commonly used for the induction and maintenance of general anesthesia because it is a rapid-onset and short-acting hypnotic agent. Moreover, propofol is known to have an antiemetic effect and TIVA with propofol is effective to reduce the incidence of PONV (11). General anesthesia with volatile anesthetics is largely responsible for PONV and avoidance of volatile anesthetics alone reduced the incidence of PONV by 19% (5). In our study, propofol-based TIVA reduced the incidence of PONV, even in ESRD patients.

For patient characteristics, female sex was considered the most important risk factor for PONV in several previous reports (3, 18–20). In these articles, female patients suffered from PONV three times more often than male patients. This may be due to hormone status, since this difference between the sexes begins at puberty. Nevertheless, the menstrual cycle does not have an impact on the occurrence of PONV (21). Although the mechanism of high PONV incidence in females remains unclear, our study showed the same results with female sex increasing the incidence of PONV.

Anesthetic duration is believed to increase PONV (18, 22). Correlation between anesthetic duration and PONV was same in our study. Some studies demonstrated that sufficient intravenous fluid administration might effectively prevent PONV (23, 24). But, there was no difference according to the amount of crystalloid infusion in our study.

There are some limitations to our study. First, similar to other retrospective studies, the data were incomplete so it may have introduced unrecognized bias into the results. In addition, some baseline characteristics of the two groups were significantly different. It might cause selection bias. Second, we did not evaluate patient-specific risk factors such as smoking status, history of motion sickness, and previous PONV. Despite these factors being strongly associated with PONV, our study did not reveal a correlation. Third, we analysed the data only up to POD 1 because of most patients were discharged at POD 2; therefore, we did not compare subsequent days. Finally, nausea is a subjective symptom so the collected data relied on patient answers.

In conclusion, propofol-based TIVA is the most influential factor in decreasing PONV after peripheral vascular surgery in ESRD patients. Additionally, female sex and anesthetic duration might be increasing factors of PONV. Considering the strong prevalence of PONV in ESRD patients, anesthesiologists can apply propofol-based TIVA as an alternative to anesthesia with volatile anesthetics.

## Data Availability

The raw data supporting the conclusions of this article will be made available by the authors, without undue reservation.

## References

[1] Weibel S Rücker G Eberhart LH Pace NL Hartl HM Jordan OL Drugs for preventing postoperative nausea and vomiting in adults after general anaesthesia: a network meta-analysis. Cochrane Database Syst Rev. (2020) 10(10):Cd012859. 10.1111/anae.15295 33075160 PMC8094506

[2] Gan TJ Sloan F de L Dear G El-Moalem HE Lubarsky DA. How much are patients willing to pay to avoid postoperative nausea and vomiting? Anesth Analg. (2001) 92(2):393–400. 10.1213/00000539-200102000-00022 11159239

[3] Koivuranta M Läärä E Snåre L Alahuhta S. A survey of postoperative nausea and vomiting. Anaesthesia. (1997) 52(5):443–9. 10.1111/j.1365-2044.1997.117-az0113.x 9165963

[4] Apfel CC Stoecklein K Lipfert P. PONV: a problem of inhalational anaesthesia? Best Pract Res Clin Anaesthesiol. (2005) 19(3):485–500. 10.1016/j.bpa.2005.03.001 16013696

[5] Apfel CC Korttila K Abdalla M Kerger H Turan A Vedder I A factorial trial of six interventions for the prevention of postoperative nausea and vomiting. N Engl J Med. (2004) 350(24):2441–51. 10.1056/NEJMoa032196 15190136 PMC1307533

[6] Asgari MR Asghari F Ghods AA Ghorbani R Motlagh NH Rahaei F. Incidence and severity of nausea and vomiting in a group of maintenance hemodialysis patients. J Renal Inj Prev. (2017) 6(1):49. 10.15171/jrip.2017.09 28487872 PMC5414519

[7] Morsch CM Gonçalves LF Barros E. Health-related quality of life among haemodialysis patients–relationship with clinical indicators, morbidity and mortality. J Clin Nurs. (2006) 15(4):498–504. 10.1111/j.1365-2702.2006.01349.x 16553764

[8] Feldman HI Kobrin S Wasserstein A. Hemodialysis vascular access morbidity. J Am Soc Nephrol. (1996) 7(4):523–35. 10.1681/ASN.V74523 8724885

[9] Shemesh D Raikhinstein Y Orkin D Goldin I Olsha O. Anesthesia for vascular access surgery. J Vasc Access. (2014) 15(7_suppl):38–44. 10.5301/jva.5000233 24817453

[10] Eger EI Koblin DD Bowland T Ionescu P Laster MJ Fang Z Nephrotoxicity of sevoflurane versus desflurane anesthesia in volunteers. Anesth Analg. (1997) 84(1):160–8. 10.1213/00000539-199701000-00029 8989018

[11] Sneyd J Carr A Byrom W Bilski A. A meta-analysis of nausea and vomiting following maintenance of anaesthesia with propofol or inhalational agents. Eur J Anaesthesiol. (1998) 15(4):433–45. 10.1097/00003643-199807000-00009 9699101

[12] Von Elm E Altman DG Egger M Pocock SJ Gøtzsche PC Vandenbroucke JP. The strengthening the reporting of observational studies in epidemiology (STROBE) statement: guidelines for reporting observational studies. Ann Intern Med. (2007) 147(8):573–7. 10.7326/0003-4819-147-8-200710160-00010 17938396

[13] Watcha MF White PF. Postoperative nausea and vomitingits etiology, treatment, and prevention. Anesthesiology. (1992) 77(1):162–84. 10.1097/00000542-199207000-00023 1609990

[14] Apfel C Heidrich F Jukar-Rao S Jalota L Hornuss C Whelan R Evidence-based analysis of risk factors for postoperative nausea and vomiting. Br J Anaesth. (2012) 109(5):742–53. 10.1093/bja/aes276 23035051

[15] Abu Farsakh N Roweily E Rababaa M Butchoun R. Evaluation of the upper gastrointestinal tract in uraemic patients undergoing haemodialysis. Nephrol Dial Transplant. (1996) 11(5):847–50. 10.1093/oxfordjournals.ndt.a027411 8671907

[16] Chong VH Tan J. Prevalence of gastrointestinal and psychosomatic symptoms among a sian patients undergoing regular hemodialysis. Nephrology. (2013) 18(2):97–103. 10.1111/nep.12000 23078158

[17] Barth C Boer W Garzoni D Kuenzi T Ries W Schaefer R Characteristics of hypotension-prone haemodialysis patients: is there a critical relative blood volume? Nephrol Dial Transplant. (2003) 18(7):1353–60. 10.1093/ndt/gfg171 12808173

[18] Cohen MM Duncan PG DeBoer DP Tweed WA. The postoperative interview: assessing risk factors for nausea and vomiting. Anesth Analg. (1994) 78(1):7–16. 10.1213/00000539-199401000-00004 8267183

[19] Apfel CC Läärä E Koivuranta M Greim C-A Roewer N. A simplified risk score for predicting postoperative nausea and vomiting conclusions from cross-validations between two centers. J Am Soc Anesthesiologists. (1999) 91(3):693. 10.1097/00000542-199909000-00022 10485781

[20] Stadler M Bardiau F Seidel L Albert A Boogaerts JG. Difference in risk factors for postoperative nausea and vomiting. J Am Soc Anesthesiologists. (2003) 98(1):46–52. 10.1097/00000542-200301000-00011 12502978

[21] Eberhart L Morin A Georgieff M. The menstruation cycle in the postoperative phase. Its effect of the incidence of nausea and vomiting. *Anaesthesist*. (2000) 49(6):532–5. 10.1007/s001010070095. 10928257

[22] Apfel C Greim C Haubitz I Goepfert C Usadel J Sefrin P A risk score to predict the probability of postoperative vomiting in adults. Acta Anaesthesiol Scand. (1998) 42(5):495–501. 10.1111/j.1399-6576.1998.tb05157.x 9605363

[23] Maharaj CH Kallam SR Malik A Hassett P Grady D Laffey JGJA Preoperative intravenous fluid therapy decreases postoperative nausea and pain in high risk patients. Anesth Analg. (2005) 100(3):675–82. 10.1213/01.ANE.0000148684.64286.36 15728051

[24] Holte K Klarskov B Christensen DS Lund C Nielsen KG Bie P Liberal versus restrictive fluid administration to improve recovery after laparoscopic cholecystectomy: a randomized, double-blind study. Ann Surg. (2004) 240(5):892. 10.1097/01.sla.0000143269.96649.3b 15492573 PMC1356497

